# Dynamic compression locking system versus multiple cannulated compression screw for the treatment of femoral neck fractures: a comparative study

**DOI:** 10.1186/s12891-020-03259-5

**Published:** 2020-04-13

**Authors:** Dong-Ping Shu, Ya-Ping Xiao, Ming-Jian Bei, Tao Ji, Yong-Jun Peng, Bing Ma, Shao-Gang Li

**Affiliations:** 1 Department of Orthopedic Surgery, CR & WISCO General Hospital, Affiliated to Wuhan University of Science and Technology, No. 209 Yejin Road, Wuhan, Hubei Province China; 2 Department of Orthopedic Surgery, Emergency General Hospital, Beijing, China

**Keywords:** Femoral neck fractures, Internal fixation, Surgery, Compression locking plate, Aged

## Abstract

**Background:**

Femoral neck fractures are one of the problems in clinical treatment. The prognosis is uncertain. Currently, No internal fixation method is superior to other internal fixation methods in the treatment of femoral neck fractures. Therefore, the internal fixation system needs to be further explored. The aim of this study was to compare clinical outcomes of femoral neck dynamic compression locking system (DCLS) and multiple cannulated compression screws(MCCS) in the treatment of femoral neck fractures.

**Methods:**

A prospective analysis of 54 cases of femoral neck fractures treated with either a DCLS (*n* = 28) or MCCS (*n* = 26) was conducted between December 2015 and November 2017 in authors’ hospitals. The perioperative and postoperative parameters of the two groups were recorded and evaluated.

**Results:**

Fifty-four patients were followed up for 24–47 months. The etiology was caused by a fall. There was no significant difference in follow-up time, operation time, incision length, surgical blood loss, the incidence of perioperative and postoperative healing complications, and mobility in the two groups (all *P* > 0.05). The Harris score, fracture healing time, femoral neck shortening, partial weight-bearing time and complete weight-bearing time were significantly better in the DCLS group than in the MCCS group (all *P* < 0.05). The fracture healing rate in the DCLS group was higher than that in the MCCS group.

**Conclusions:**

The DCLS and MCCS might be equally effective in terms of operation time, incision length, surgical blood loss, the incidence of perioperative and postoperative healing complications, and mobility in the treatment of femoral neck fractures. However, the DCLS is superior to the MCCS in Harris score, fracture healing time, femoral neck shortening, weight-bearing time and fracture healing rate. So, DCLS deserves further study.

## Background

Femoral neck fractures are one of the most common fractures in the elderly and will reach 63 million by 2050, about half of which will occur in Asia, which seriously affects the quality of life of elderly patients [1]. For elderly patients with displaced femoral neck fractures (Garden III/IV), Less active individuals may receive a hemiarthroplasty, while more active individuals are treated with total hip arthroplasty [2]. The main complications of arthroplasty are periprosthetic dislocation, infection, and revision surgery, which potentially impact morbidity and quality of life and may contribute to mortality [3]. The treatment of elderly non-displaced femoral neck fractures (Garden I/II) is still controversial [4]. Given that non-surgical treatment of fractures is prone to re-displacement, the re-displacement is as high as 33 to 44%, and the fracture healing rate is only 44.38% [4]. At present, elderly patients with non-displaced femoral neck fractures tend to be surgically treated [5].

The choice of treatment requires comprehensive consideration of factors such as fracture classification, patient physical status, pre-injury activity level, patient willingness, and doctor habits [6]. Among many internal plants used for femoral neck fractures, multiple cannulated compression screws (MCCS) has the advantages of small surgical trauma, short surgical time, and reliable fixation. Three parallel cannulated screws in an inverted triangle configuration have the advantages of strong grip and sliding pressure, which can significantly increase the healing rate of fractures and reduce postoperative complications [7]. However, there were reports in the literature that the use of MCCS for Garden I and II fractures had a higher rate of surgical revision [8].

Currently, there is no one internal fixation for the treatment of femoral neck fractures that shows superiority over other internal fixations [9]. Therefore, the internal fixation system needs to be further explored. DCLS is a new method for femoral neck fracture fixation, which is a combination of the MCCS and dynamic hip screws. The main features are as follows. ①The positions of the three parallel compression screws are distributed on the triangular protuberance of the safety cross section of femoral neck with high bone density, which conforms to the “cortical support” principle and has the characteristics of screw dispersion and maximum holding force [10]. ②Controlled axial uniform dynamic compression can effectively control excessive shortening of femoral neck. ③The three screws press the fracture end parallelly to axial direction of femoral neck through lateral locking plate postoperatively, which can provide axial and uniform positive pressure for accommodate collapse. ④The frame configuration is stable and has good resistance to shear and torsion. ⑤The even pressure intraoperatively can make the fracture ends well aligned and reduce the infiltration of synovial fluid, which could be conducive to callus formation and fracture healing. Biomechanical experiment and finite element analysis confirmed that DCLS had good fixed stability and biomechanical conductivity without stress shielding, and was conducive to fracture healing [11].

Therefore, in the present study, based on the aforementioned considerations, we prospectively collected and evaluated the clinical outcomes of Garden I, type II and III femoral neck fractures surgically treated by internal fixation with either a DCLS or MCCS. The objectives were to compare the clinical outcomes of DCLS and MCCS in the treatment of Garden I, II, and III femoral neck fractures and to investigate the advantages and disadvantages of the DCLS.

## Methods

### Patients

Inclusion criteria are as follows: ① Garden I, II, and III femoral neck fractures; ② Patients were treated with DCLS (Suzhou Kangli Orthopedic Medical Equipment Co., Ltd., Jiangsu, China) or MCCS; ③Patients can walk autonomously before injury; ④The follow-up time was at least 2 years. Exclusion criteria are as follows: ①Pathological fractures other than osteoporosis; ②Combined with other limb fractures; ③Complicated with severe medical diseases and surgical contraindications.

**Fig. 1:**
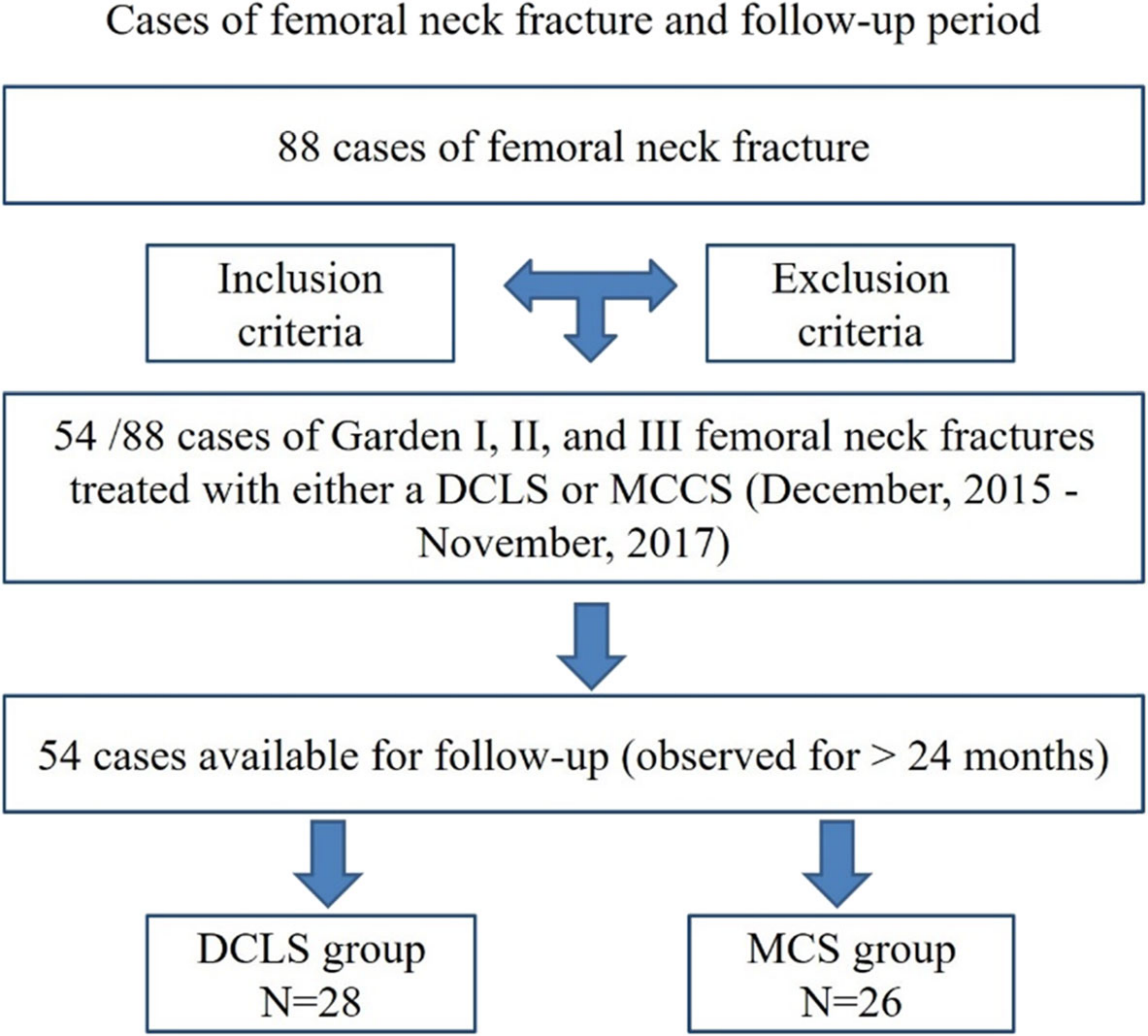
Cases of femoral neck fracture and follow-up period

From December 2015 to November 2017, 88 patients with femoral neck fractures were surgically treated. According to the inclusion and exclusion criteria, a total of 54 patients were included in this study (Fig. 1). According to different surgical methods, they are divided into DCLS group (*n* = 28) and MCCS(*n* = 26) group. This study was approved by the ethics committee of our hospitals. Informed consents for all clinical details and images publication were taken from all the patients.

There were no significant differences in the baseline characteristics, such as age, gender, left and right sides, body mass index (BMI), Singh index, injury-to-operation time, number of osteoporosis, and Garden classification [12] between the two groups (Table 1, all *P* > 0.05), which were comparable. All patients were injured due to falls.

**Table 1:** Comparison of general information between DCLS group and MCS group

Group	Cases	Gender (female/male)	Age(Year)	Osteoporosis (Yes /No)	Garden typing (I/II/III)	Side (left /right)	BMI	Singh index	injury-to-operation time(Day)	Cause of injury (falls)
DCLS	28	21/7	65.8 ± 10.3	19/9	3/6/19	18/10	23.0 ± 5.4	2.1 ± 0.8	2.5 ± 0.9	28
MCCS	26	20/6	67.2 ± 10.2	18/8	2/7/17	18/8	24.1 ± 5.8	2.4 ± 0.9	2.9 ± 1.0	26
t/χ^2^	–	0.027	−0.495	0.012	0.314	0.148	−0.721	−1.403	−1.428	–
*P*	–	0.869	0.625	0.914	0.855	0.700	0.474	0.167	0.159	–

### Surgical procedures

The patient was placed supine on an orthopedic traction table. After the C arm x-ray machine confirmed that the fracture was in a good reduction position, conventional sterilization was performed. The affected limb was slightly abducted and internally rotated. A longitudinal incision about 4 cm was made under the greater trochanter. In the DCLS group, the surgeon inserted three parallel cannulated compression screws in the front-upper, rear and inferior of femoral neck in order to form an unequal triangle. At the end of screws insertion period, the three screws were pressed uniformly, precisely, and strongly in order. Finally, the locking screw caps were placed in the small side plate to make the screws as a whole [10]. In the MCCS group, three parallel guide needles were inserted into the femoral head along the longitudinal axis of femoral neck in a triangular configuration. After the screw position was proper, three cannulated screws were screwed and finally pressed evenly. Note that the screw entry point should not be lower than the level of the lesser trochanter to reduce stress concentration. The distal thread should completely pass through the fracture line. The top of the screw should reach 5–10 mm below the femoral head cartilage and the screw should be as close to the cortex as possible [13].

### Perioperative management

Antibiotics were administered 0.5 h before surgery. Patients were encouraged to perform non-weight-bearing functional exercises after 1–2 days postoperatively. Postoperative ankle flexion and extension exercises and routine use of low molecular heparin 4000 IU subcutaneously were applied to prevent deep vein thrombosis for a mean of 1 month after surgery. Patients with osteoporosis were treated with calcium tablets and diphosphates. Partial weight bearing can be performed according to the recovery of the affected limb. About 3 months after surgery, whether walk with full weight can be decided according to bone healing. X-ray reexamination was performed within 3 days after operation. X-ray follow-up was performed monthly for the first 6 months after operation, followed by every 3 months thereafter, and every 6 months after 1 year.

### Outcome measurement

All clinical data for operative time, incision size, surgical blood loss, incidence of postoperative and bone healing complications, Harris scoring, fracture healing time and rate, femoral neck shortening, weight-bearing time, mobility were recorded and assessed. Nonunion was judged according to the criteria described by Dhar et al. [14]. Femoral head necrosis was judged according to the standard of Slobogean et al. [15]. According to Slobogean et al. [16] definition of femoral neck shortening, the difference between measured value on the affected side and the normal side of pelvic orthotopic x-ray film was assessed as femoral neck shortening. Femoral neck horizontal shortening was measured from the inside of femoral head to the outside of greater trochanter. Femoral neck vertical shortening was measured from the upper edge of femoral head to greater trochanter. Hip function was assessed according to Harris scoring criteria [17]. It was scored from 4 aspects of pain, function, deformity, and exercise, with a perfect score of 100, of which 90–100 were excellent, 80–89 were good, and 70–79 were better, < 70 points are poor. Mobility evaluation was based on a 4-level walking classification, that is, walking without any assistive tools, walking with crutches, walking with walking aids, and walking with wheelchairs [18].

### Statistical analysis

Continuous data were expressed as mean ± standard deviation and analyzed using Independent-Samples T Test. Categorical data were expressed as absolute numbers or percentages and analyzed using χ^2^ test. Statistical analyses were performed with SPSS 19.0 software (SPSS Inc., USA). A *P* < 0.05 was considered statistically different.

## Results

All 54 patients were followed up for 24–47 months. There were no significant differences in follow-up time, surgical time, intraoperative blood loss, and surgical incision length between the two groups (all *P* > 0.05, Table 2).

**Table 2:** Comparison of general results, femoral neck shortening, and fracture healing between DCLS group and MCCS group

Group	Follow-up time (Months)	operation time (Minutes)	Surgical blood loss (ml)	Incision length (cm)	Femoral neck shortening		healing time (Months)	Fracture healing rate (%)
					horizontal (mm)	Vertical (mm)		
DCLS	35.7 ± 6.4	58.7 ± 9.0	56.8 ± 9.5	4.2 ± 0.53	4.4 ± 1.45	6.8 ± 2.27	3.3 ± 0.50	92.9
MCCS	36.7 ± 5.7	59.0 ± 11.4	56.2 ± 9.2	4.3 ± 0.57	7.7 ± 1.23	8.9 ± 2.28	4.1 ± 0.76	88.5
t	−1.569	−0.740	1.366	−3.826	−55.195	−20.519	−27.702	–
*P*	0.574	0.459	0.172	0.000	0.000	0.000	0.000	–

Femoral neck shortening occurred in both groups after surgery. The DCLS shortening in the horizontal and vertical direction were significantly lower than those in the MCCS group (All *P* = 0.000, Table 2).

**Fig. 2:**
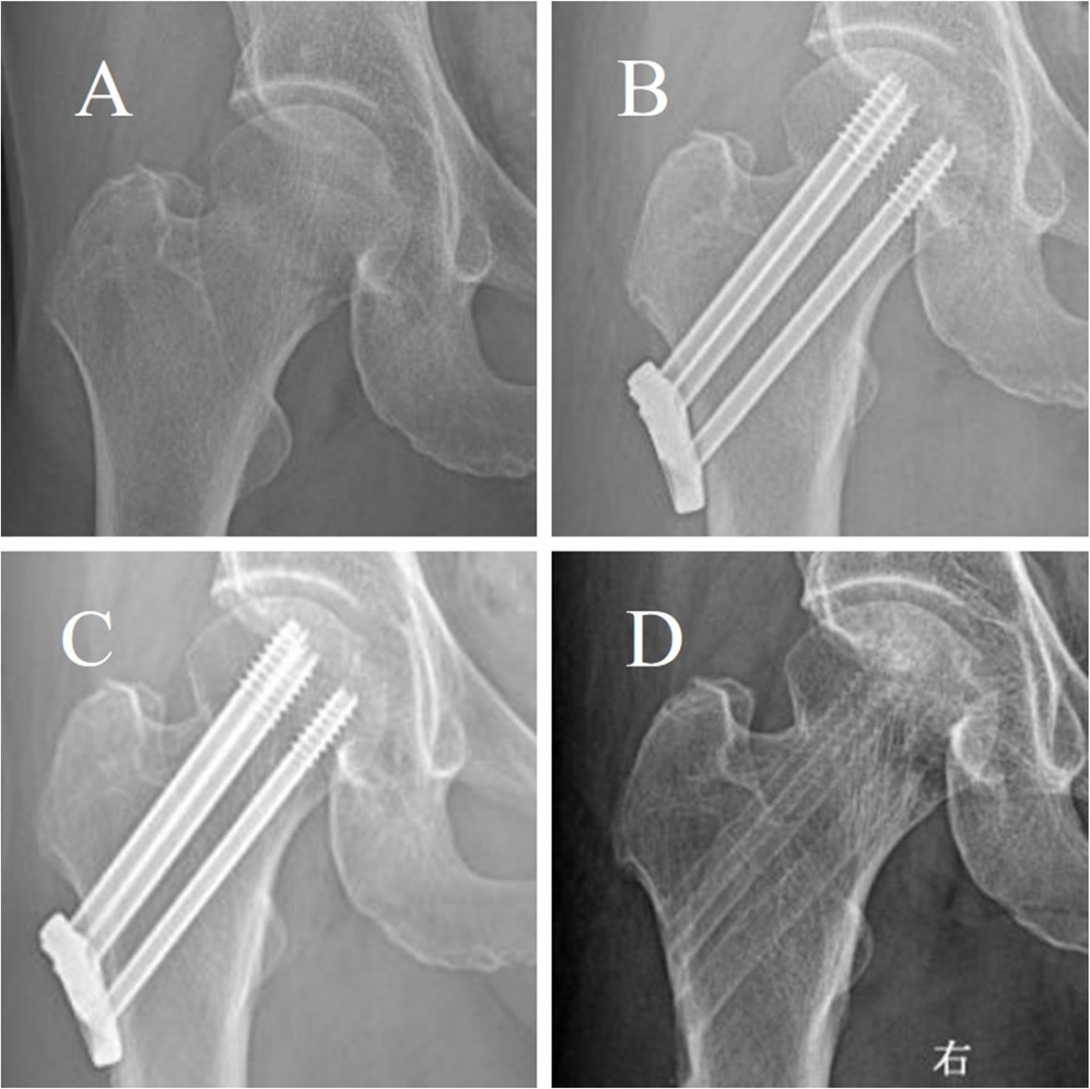
Typical radiograsphs of a displaced femoral neck fracture in 62-year-old female treated by closed reduction and fixed by DCLS and later removd without complaints. **a**: Pre-operation **b**: Post-operation **c**: Twenty-three months postoperatively **d**: Post removal of the DCLS at twenty-three months postoperatively

**Fig. 3:**
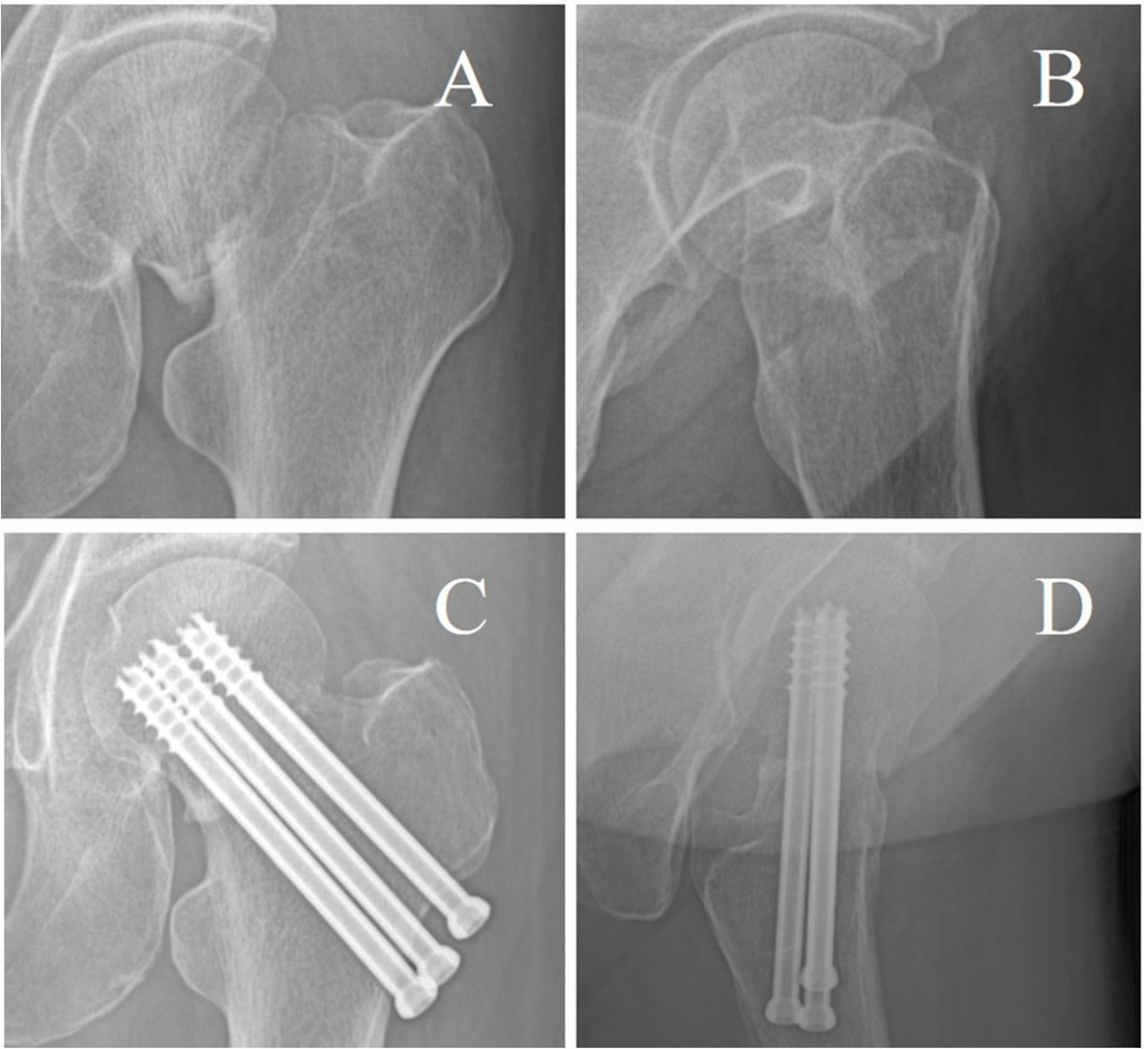
Typical case of three hollow screws for femoral neck fractures. **a**: Preoperative anteroposterior X-ray radiography **b**: Preoperative lateral X-ray radiography **c**: Postoperative anteroposterior X-ray radiography **d**: Postoperative lateral X-ray radiography

Fracture healing rate of 92.9% (26/28) in the DCLS group (Fig. 2) was significantly higher than that of 88.5% (23/26) in the MCCS group (Fig. 3). The bone healing time of 3.3 ± 0.50 months (range, 2.3–4.3 months) in the DCLS group was significantly shorter than that of 4.1 ± 0.76 months (range, 3.1–6.2 months) in the MCCS group (*P* = 0.000, Table 2).

The partial weight-bearing time and full weight-bearing time in the DCLS group were significantly shorter than those in the MCCS group (*P* = 0.000, respectively, Table 3). At the last follow-up, the Harris score of the DCLS group was significantly higher than that of the MCCS group (*P* = 0.000, Table 3). The Harris score in the DCLS group was significantly higher than in the MCCS group (*P* = 0.000), and the excellent rate in the DCLS group was higher than in the MCCS group, but there was no significant difference in Harris rating between the two groups (*P* > 0.05, Table 3). All patients in the DCLS and MCCS group could walk autonomously before injury. Patients maintaining preoperative mobility (96.4%) at the last follow-up in the DCLS group was higher than that (88.5%) in the MCCS group (Table 3).

**Table 3:** Comparison of postoperative functional recovery between DCLS group and MCCS group

Group	partial weight-bearing time (Days)	full weight-bearing time (Months)	Harris rating			Harris score	Excellent and good rate (%)	mobility	
			excellent	good	poor			Maintain preoperative activities	Down one level
DCLS	13.7 ± 7.3	3.5 ± 0.70	16	10	2	91.8 ± 8.8	92.9	27 (96.4%)	1 (3.6%)
MCCS	36.9 ± 10.9	5.6 ± 1.83	15	8	3	88.1 ± 9.5	88.5	23 (88.5%)	3 (11.5%)
t/χ^2^	−54.236	−33.211		2.599		13.757	–		1.248
*P*	0.000	0.000		0.273		0.000	–		0.264

Non-union occurred in 2/28 (7.1%) patients in the DCLS group (Table 4). One of them, 66 years old, Garden III, had hip pain and discomfort at 24 months after surgery. He underwent surgery for internal fixation removal. Postoperative hip joint pain still existed. MRI examination revealed nonunion. At the last follow-up 45 months after surgery, the hip-preserving treatment continued. Another one, 84 years old, Garden III, had hip pain when walking at 7 months after surgery, and X-rays revealed non-union. Hemi-hip replacement was performed. In the MCCS group, nonunion occurred in 2/26 cases (7.7%), of which 1 case, 66 years old, Garden III was failed when walking after 7 months, and bone cement hemi-hip replacement was performed later. Another one, 76 years old, Garden III, was revealed nonunion by X-ray re-examination at 13 months after surgery and biological hemiarthroplasty was performed. Femoral head necrosis (3.8%) in the MCCS group was reported in One, 71 years old, and type II Garden fracture female with osteoporosis. After 15 months of surgery, MRI examination showed necrosis and total hip replacement was performed. There was no significant difference in the incidence of bone healing complications between the two groups (Table 4). None of the patients in the two groups had perioperative complications such as wound infection and deep vein thrombosis etc. (Table 4).

**Table 4:** Comparison of complications between DCLS group and MCCS group

Group	Healing complications			Perioperative complications
	Non-union	Femoral head necrosis	None	
DCLS	2 (7.1%)	0 (0%)	26 (92.9%)	0
MCCS	2 (7.7%)	1 (3.8%)	23 (88.5%)	0
χ^2^		1.111		–
*P*		0.574		–

## Discussion

The operation time, length of incision, surgical blood loss, perioperative complications and incidence of bone healing complications were not statistically different between DCLS group and MCCS group, suggesting that the surgical incision in both groups was small with small trauma and less blood loss during the surgery, so both surgeries could be minimally invasive with less complications. The DCLS group had fewer healing complications than did the MCCS group, but there was no statistical difference. The Harris score, fracture healing time, femoral neck shortening, partial weight-bearing time and complete weight-bearing time at the last follow-up were significantly better in the DCLS group compared with the MCCS group. The fracture healing rate and mobility were better in the DCLS group than in the MCCS group, which suggests that DCLS, compared with MCCS, could increase fracture healing rate, improve patients’ mobility and hip function, accelerate earlier bone healing and prevent excessive shortening of femoral neck. So patients in the DCLS group can carry out weight-bearing activities earlier, and have the better quality of life.

Femoral neck fractures are one of the problems in clinical treatment. The prognosis is uncertain. Nonunion and femoral head necrosis are recognized as serious complications after internal fixation. The type of femoral neck fracture and improper treatment are considered to be the main factors of nonunion and femoral head necrosis [19]. Garden classification is the mainstream classification system for femoral neck fractures and guides clinical treatment. Decisions on the treatment of displaced (unstable) fractures (Garden III and IV) in young patients are still controversial. Surgical methods include closed/open reduction internal fixation, hemi-hip replacement, and total hip replacement [20]. Stable fractures (Garden I and II) tend to be treated with internal fixation.

Femoral neck fractures, no matter what treatment method is selected, have a significant impact on the living quality of patients and bring a large economic burden to society [21]. Although internal fixation has a higher incidence of postoperative revision rate, complications, nonunion, delayed bone healing, and poor function in the treatment of undisplaced femoral neck fractures for super-aged patients [22], internal fixation still is currently preferred for Garden I and II femoral neck fractures [6]. However, there is still no consensus on which internal fixation method can better maintain the stability of fractured ends, promote fracture healing, and avoid and reduce complications such as femoral head necrosis, nonunion, and internal fixation failure [23].

Three cannulated screws can exert pressure on the fracture end and promote fracture healing. In addition, they occupy a relatively small area of femoral neck, and have less interference with blood flow for femoral head and neck. The triangular distribution can form a three-dimensional frame with bone tissue, which can improve stress against the rotation of femoral head. It can enhance compressive stress intraoperatively and postoperatively between fracture ends, which could promote closer contact between fracture ends and be conducive to fracture healing. However, because the three cannulated screws are not related to each other; the position of the screws are easily affected by subjective and objective factors of the operator. So its resistance to vertical shear and torsion is relatively poor, which can lead to loosening and re-displacement of fracture end, femoral head necrosis, nonunion, and femoral neck shortening [24, 25]. And in the process of healing, lack of sustained and effective solid support will affect the rehabilitation training of the affected limb and growth of the fractures [9].

Although dynamic hip screw can provide better angular stability and sliding compression, its anti-rotational stability is poor, especially when the hip screw is screwed, which can easily cause poor alignment of the femoral head and neck [5]. Furthermore, dynamic hip screw require large soft tissue exposure, and hip screw insertion damages the cancellous bone of femoral head and neck and destroy its blood supply, which affects the healing of femoral neck fractures.

DCLS consists of three conventional hollow lag screws, one femur lateral plate with locking screw holes and three locking tail caps. During the operation, three conventional hollow lag screws were inserted into femoral neck through the lateral plate to perform static compression and fixation on the fracture end, and then the three locking tail caps were screwed into the locking thread hole on the lateral plate to achieve screw locking. In the process of fracture healing, because the lateral plate is not fixed with the femur, there is good dynamic pressure between the fracture ends under external force loading. Therefore, this design can simultaneously achieve static and dynamic pressure action, to meet the necessary conditions for fracture healing. At the same time, the tail-cap locking design of the system makes the three screws become an integrated rigid frame structure, and the screws can support each other. Therefore, DCLS combines the advantages of MCCS and dynamic hip screw, which can not only improve strong, uniform and accurate compression of the fracture section intraoperatively, but also have stable frame structure to stabilize the broken end of the fracture and controlled dynamic compression to prevent excessive shortening of femoral neck. So it can obtain good initial and continuous stability to prevent displacement of the fracture ends and help fracture healing. Early biomechanical experiment of human corpses showed that DCLS, compared with MCCS, has better biomechanical stability, stronger compressive and torsional resistance [11].

The tail-cap locking design of DCLS makes the three screws become an integrated rigid frame structure, and the screws can support each other. The load can be evenly distributed among three screws and applied to the lateral plate. On the one hand, The structure can be more even effectively combat stress, bending stress, tensile stress and rotation. On the other hand, although the system does not have the traditional angular stability design, the vertical shear stress can be transferred to the lateral plate by screws, and then the counterforce between the lateral plate and the femoral lateral bone cortex can be used to resist the vertical shear stress. This is completely different from traditional three cannulated lag screws, whose resistance to shear force can only be realized by relying entirely on the limited contact between the screw end cap and the lateral bone cortex. The contact area of this contact is much smaller than that of the lateral plate and the bone cortex in DCLS. So the angular stability of the three cannulated lag screws was worse than that of DCLS. In biomechanical experiments, when the lateral loading was 400 N in the horizontal compressive loading test, the compressive stiffness (4324 ± l 234) N/mm of DCLS was significantly larger than that of the three cannulated lag screws (3020 ± 855) N/mm (*P* = 0.0050) [11]. The torsional stiffness of DCLS (11.45 ± 4.95) N·m/° was significantly larger than that of the three cannulated lag screws (6.53 ± 4.83) N·m/° when the torsional load was 2.5 N m/° (*P* = 0.0423) [11].

At present, three cannulated screws are commonly used for internal fixation of stable femoral neck fractures, but many studies have significant differences in the position distribution and clinical overcomes [26]. The three cannulated screws have large differences in torsion resistance and fracture end stability [27]. The instability of the fracture end is not conducive to fracture healing. Weil et al. [28] used three cannulated screws in an inverted triangle to treat 41 cases of femoral neck fractures, 71% of them had a significant femoral neck horizontal shortening greater than 5 mm, and 25% of them had severe shortening greater than 10 mm. Significant shortening occurred in 43% of patients in the vertical direction, and severe shortening occurred in 17% of the patients. Screw pullout greater than 5 mm occurred in 41% of patients. 7 cases required late hip replacement. Gupta et al. [29] studied hollow cancellous screws for femoral neck fractures for up to 4 years. The imaging healing time was 7.1 months, the healing rate was 82.22%, the osteonecrosis rate was 6.67%, and the Harris Hip Score was 88.65. Manohara et al. [30] studied cancellous screw fixation for undisplaced femoral neck fractures in elderly patients, Of the 96 patients followed up for a mean of 39 months, 8/96 (8.3%) underwent revision surgery for femoral head avascular necrosis (5/96, 5.2%) or non-union/implant failure (3/96, 3.1%). Overall, 30/96 (31.3%) patients had a decrease in their mobility status. Chen et al. [31] studied patients with femoral neck fractures treated with the dynamic hip system blade or MCCS for an average follow-up of 27 months. No statistically significant differences in the rates of nonunion (4.5% vs. 0) and femoral head avascular necrosis (9.1% vs. 7.1%) were observed. 15.9% of patients reported a femoral neck shortening greater than 10 mm. Other study has found that for femoral neck fractures treated with three hollow screws, nonunion and osteonecrosis were 42 and 17% in the displaced fracture group and 6 and 4% in the non-displaced fracture group [32].

However, this study had following limitations. The number of cases was relatively small. It was a single-center prospective study and has not been completely randomized and double-blind. The results may be biased. Therefore, this study needs to be verified by further multicenter, randomized, controlled, double-blind clinical trials.

## Conclusions

The DCLS and MCCS might be equally effective in terms of operation time, incision length, surgical blood loss, incidence of perioperative and postoperative healing complications, and mobility in the treatment of femoral neck fractures. However, the DCLS is superior to the MCCS in Harris score, fracture healing time, femoral neck shortening, weight-bearing time and fracture healing rate. Thus, further studies are warranted to assess the effect of DCLS in the treatment of femoral neck fractures.

## Data Availability

The datasets used and analysed during the current study are available from the corresponding author on reasonable request.

## Abbreviations

DCLS: Dynamic compression locking system
MCCS: Multiple cannulated compression screw
BMI: Body mass index
